# Effects of Mixture Proportions and Molding Method on the Performance of Pervious Recycled Aggregate Concrete

**DOI:** 10.3390/ma17215138

**Published:** 2024-10-22

**Authors:** Haifeng Wei, Lixing Yan, Caifeng Lu, Zhihong Wen, Ye Yang, Chunhao Lu, Qingsong Zhou

**Affiliations:** 1 China Communications Construction Company Limited, Beijing 100010, China; 2 Jiangsu Key Laboratory of Disaster Impact and Intelligent Prevention in Civil Engineering, School of Mechanics and Civil Engineering, China University of Mining & Technology, Xuzhou 221116, China; 3 Building Materials Engineering Laboratory, Department of Architecture, Graduate School of Engineering, The University of Tokyo, Tokyo 113-8654, Japan

**Keywords:** pervious concrete, demolished concrete waste, recycled coarse aggregate, permeability coefficient, compressive strength

## Abstract

The use of pervious concrete pavement systems with recycled aggregates is a sustainable and innovative solution to major urbanization challenges such as repurposing construction waste, alleviating urban waterlogging, and reducing heat-island effects. This study aims to investigate the effects of mixture proportions and molding methods on the performance of pervious recycled aggregate concrete (PRAC). To this end, the coarse aggregate size (4.75~9.5 mm, 9.5~16 mm, and 16~19 mm), the molding method (layered insertion-tamping and vibration molding with vibration times of 5 s, 10 s, or 15 s, respectively), and the replacement rate of recycled coarse aggregate (RCA) (0%, 30%, 50%, and 100%, respectively) are considered. The results reveal that the addition of RCA to permeable concrete weakens its permeability. However, the compressive strength of PRAC reaches its maximum value when the RCA replacement rate is 50%. A larger aggregate particle size (16~19 mm) enhances the compressive strength of PRAC, yet decreases the permeability of PRAC. By using vibration molding to fabricate PRAC, an extension to the vibration duration increases the compressive strength, yet concurrently decreases the permeability. Based on the compressive strength and permeability coefficient of PRAC, the optimal mixture proportions and molding method are suggested.

## 1. Introduction

In recent years, with the continuous advancement of national industrialization and urbanization processes, significant changes have occurred in the hydrological cycle due to the replacement of natural vegetation and the closure of soil. A reduction in permeable surfaces intensifies an imbalance in the hydrological cycle, and a significant increase in surface runoff causes overloading of rainwater drainage systems, flood disasters, and urban heat islands. To overcome these serious ecological problems, local governments have proposed a concept of “sponge cities”, and a permeable pavement system is the core of the construction of “sponge cities” [1,2,3]. Permeable pavement systems are considered to be one of the most effective solutions to mitigate surface runoff, with their unique pore structure designed to efficiently facilitate the drainage of surface water, which aids in alleviating issues related to urban waterlogging and heat-island effects. Permeable concrete (PerC) is a lightweight porous concrete composed of coarse aggregate (with less or even no fine aggregate), cement, reinforcing agents, and water, which has the characteristics of pore diameters ranging from 2 to 8 mm, voids comprising 15% to 35% of its volume, and high permeability exceeding 0.014 cm/s [4,5]. As a result, PerC is ideally suited for use as the primary layer within permeable pavement systems. It plays a crucial role in enabling the rapid infiltration of water through its interconnected pores and has been increasingly applied in practical construction projects [5,6,7].

Large amounts of construction and demolition waste (C-D waste) have been generated by rapid urbanization and economic development. In China, the annual production of C-D waste has exceeded 600 million tons [8], which has brought serious impacts to the ecological environment. China has the highest production of C-D waste but only 40% of it has been recycled [9]. In the European Union, the annual production of construction waste is approximately 900 million tons, constituting 25% to 40% of all waste generated [10]. The EU’s waste management guidelines stipulate that member states must have achieved a minimum recycling rate of 70% for the total production of C-D waste by 2020 [5]. Numerous studies have shown that recycled aggregates (RAs) generated from C-D waste can be applied to various civil engineering projects, which not only helps reduce the demand for raw materials and energy consumption but also provides promising solutions to address sustainable environmental challenges in building practices [8,10,11,12,13].

The PerC produced by using RAs as part or all the coarse aggregate is called permeable recycled aggregate concrete (PRAC). Extensive research has been conducted on PRAC, including the effects of recycled coarse aggregate (RCA) properties [14,15,16,17,18,19,20,21], sand content [21,22], water–cement ratio [22,23], basic concrete strength [24,25], and mineral admixtures [26,27,28,29,30] on various aspects of PRAC performance. For example, Guneyisi et al. [14] and Sata et al. [15] have shown that an increase in RCA content can lead to a decrease in the mechanical properties of PRAC. However, Barnhouse et al. [16] found that the performance of PerC is not significantly affected by the addition of RCA. Zaetang et al. [17] found that a slightly higher RCA substitution rate than 50% increases the compressive strength and significantly improves the wear resistance of PRAC, while a 100% substitution rate reduces the compressive strength but also improves the wear resistance. The study conducted by Chen et al. [18] indicated that pre-wetting RCA for 10 min can enhance the compressive strength of PRAC, and a 30% replacement rate of RCA is the most effective in improving both the compressive and tensile strength of PRAC. However, it is noteworthy that the porosity and permeability coefficient were observed to be the highest under the condition of a 100% replacement rate of RCA, which contradicts the research conclusion of Park et al. [19], where the maximum permeability coefficient of PRAC was achieved with a 50% RCA replacement rate. In a separate study, Yuan et al. [20] investigated the impact of the particle size distribution of RCA on the performance of PRAC and observed that PRAC with a coarse aggregate particle sizes of 10–20 mm exhibited a higher permeability coefficient when the RCA replacement rate ranged from 0% to 70%. Gao’s research [21] has revealed that the particle size distribution of aggregates exerts the greatest influence on the compressive strength, permeability coefficient, and freeze–thaw resistance of PRAC, whereas the sand content has the least impact. Yan et al. [22] and Xue et al. [23] investigated the effects of the water–cement ratio, binder content, and sand content on the compressive strength and permeability of PRAC, and confirmed the optimal water–cement ratio, binder content, and sand content that resulted in excellent strength and permeability coefficients of PRAC. Li et al. [24] manufactured PRAC using RCA sourced from waste concrete of various strengths and found that its excellent compressive strength, flexural strength, and wear resistance all met the requirements of practical engineering, which aligns with the research findings of Liu et al. [25].

To enhance the mechanical properties of PRAC, it is possible to incorporate mineral admixtures or fibers. For instance, researchers [26,27,28,29] have investigated the effects of blast furnace slag powder and different types of fibers on the compressive strength and wear resistance of PRAC. Among these studies, Guo et al. [29] observed that polypropylene fibers can decrease the compressive strength of PRAC, while carbon fibers can improve it to a certain extent. Both types of fiber materials can effectively enhance tensile and wear resistance. Xue et al. [30] demonstrated that using micro silica powder instead of ordinary Portland cement in PRAC can significantly improve compressive strength and permeability coefficients.

As evident from the above, PRAC prepared by using RCA crushed from waste concrete to replace, partially or completely, natural coarse aggregates (NCA) has already been extensively studied. The performance measurement of PRAC has been found to meet practical engineering needs, confirming the feasibility of using RCA in PerC while also providing positive performance and economic benefits. However, when it comes to pervious concrete with RAs, there is still a lack of in-depth research into certain aspects such as the optimal mix ratio and molding method for PRAC. Currently, comprehensive research has been conducted on the molding methods for ordinary concrete; for PRAC, with unique characteristics, the molding methods have a greater impact on its performance. Herein, this article discusses the role of RCA as a substitute for NCA in the performance of pervious concrete, aiming to obtain PRAC with sufficient strength and permeability to meet practical engineering needs. To achieve this goal, extensive experimental studies were carried out to evaluate the effects of the recycled aggregate substitution rate, aggregate size, and molding method on the compressive strength and permeability of PRAC.

## 2. Materials and Methods

### 2.1. Materials

In accordance with the pertinent standard delineating the requirements for PRAC [31], the materials employed in this experimental investigation encompassed Portland cement, natural coarse aggregate (NCA), recycled coarse aggregate (RCA), a water-reducing agent, and water. In the pursuit of formulating pervious concrete characterized by favorable water permeability (permeability coefficient greater than 0.5 mm/s), the utilization of fine aggregates was deliberately omitted.

PRAC should adopt Portland cement or ordinary Portland cement with a strength grade of not less than 42.5 grade [31]. The cement used was PO 42.5 ordinary Portland cement, as specified in *Common Portland Cement* (GB175) [32], and the key chemical compounds and physical properties are detailed in Table 1.

The permeable surface layer of PRAC should adopt single graded aggregates of 4.75~9.50 mm or 9.50~16.0 mm, and the permeable base layer aggregates should adopt continuously graded crushed stones with a maximum particle size not exceeding 31.5 mm [31]. Both NCA and RCA fall within the particle size range of 4.75–19 mm. NCA consists of natural crushed stone conforming to the pertinent national standards [33], whereas RCA is obtained by mechanically crushing waste concrete specimens and further screening [34]. The discarded concrete components (with a strength of C30) were initially fragmented into concrete blocks through the utilization of a pile driver, then further comminuted via a jaw crusher, and finally screened employing sieves featuring square apertures of 4.75 mm and 19 mm to obtain RCA falling within the 4.75~19 mm particle size range.

Table 2 presents the main physical properties of NCA and RCA determined according to relevant standards [33,34] for comparison.

It is evident that compared with NCA, RCA exhibits a higher crushing index and water absorption rate while displaying a lower apparent density and bulk density. This phenomenon can be attributed to the adhesion of old cement slurry with high porosity on the surface of RCA, resulting in a transition layer between the coarse aggregate and old cement slurry. It is worth noting that the various physical properties of the RCA utilized in this experiment complied with the requirements outlined for Class II recycled aggregates in the relevant Chinese regulation [34,35].

A polycarboxylic acid series (ST-01A type) was used as a high-performance water-reducing agent, which met the specification requirements [36]. Common tap water from the city was used for concrete mixing [37].

### 2.2. Mixture Details

The selection of the strength grade for PerC should be comprehensively considered based on the bearing capacity requirements of the usage site and the service life. PerC with lower strength grades is suitable for sidewalks, while highways, large squares, etc., that bear large vehicles or pedestrian loads should adopt PerC with higher-strength grades. According to the Chinese industry standard *Technical Specification for Application of Pervious Recycled Aggregate Concrete* (CJJ/T 253) [31], the permeability coefficient of the PerC surface should not be less than 0.5 mm/s and the continuous porosity should not be less than 10%. Considering that excessive porosity may result in the compressive strength of PerC not meeting the usage standards, the target porosity designated in this experiment was 20% and the water–cement ratio was 0.30. The following three types of coarse aggregate particle grading were selected: 4.75 mm~9.5 mm, 9.5 mm~16 mm, and 16 mm~19 mm. The following four situations were considered for the RCA replacement ratio: 30%, 50%, 70%, and 100%. The mix proportions of PRAC adopted in the experiment are detailed in Table 3. Due to the insignificant difference in apparent density measured by the RCA and NCA used in this article (Table 2), the compositional changes per m^3^ of concrete were not considered when replacing NCA with RCA of equal weight.

In this study, the following two concrete molding methods were considered: vibration molding and layered insertion-tamping (test group number D3). The vibration molding method involved placing a concrete mixture into a mold and using a concrete vibrator to prepare concrete specimens, with vibration times set at 5 s (test group numbers 01, 02, A1, A2, B1, B2, C1, and C2, 03, 04, and 05), 10 s (test group number D1), and 15 s (test group number D2), respectively. The process of preparing concrete using the layered insertion-tamping method included dividing the wet concrete slurry into three equal layers and placing them into the mold, then using a round-headed compactor to vertically tamp each layer 20 times to ensure thorough compaction of each layer’s thickness, and finally using a scraper to flatten the surface.

As per the pertinent regulations governing recycled concrete structures [38], the mixing water volume to prepare recycled concrete should account for the additional water volume required by RCA, which can usually be treated by pre-wetting the RCA. In this experiment, the method of pre-wetting for 10 min was adopted to solve the problem of the higher water absorption of RCA [18,39].

### 2.3. Test Methods

For each group of mix proportions shown in Table 3 (a total of 14 groups), 6 cubic specimens with dimensions of 100 mm × 100 mm × 100 mm were made (84 specimens in total), of which 3 specimens were used for compressive strength testing and the other 3 specimens were used for permeability coefficient testing.

A method called “stone enveloped with cement” was adopted to mix PerC slurry [31,40], which involved firstly mixing NCA, pre-wetted RCA, and 50% mixed water for 30 s, then adding 50% cement to continue mixing for 40 s, and finally adding the remaining water and cement to continuing mixing for 1 min. PRAC specimens were made using the vibration molding method and layered insertion-tamping method, as shown in Table 3.

After 24 h from specimen production, the formwork was removed and the specimens underwent a 28-day curing process under standard conditions. Subsequently, the compressive strength and permeability coefficient were measured. The determination of compressive strength followed the relevant standard guidelines [41].

**Figure 1:**
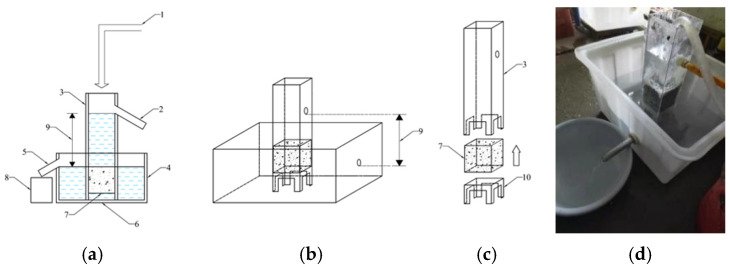
Custom-made permeability coefficient measuring device (1: water supply system; 2: overflow port of the square cylinder; 3: square cylinder; 4: water tank; 5: overflow port of water tank; 6: support; 7: concrete sample; 8: measuring cylinder; 9: water level difference; 10: test-piece base). ((**a**) details of the system; (**b**) Parameters used for calculation; (**c**) specimen insert; (**d**) actual system).

The permeability coefficient plays a pivotal role in evaluating the permeability performance of pervious concrete. This article introduces a permeability coefficient measurement device customized according to the fixed-head method, as outlined in the application regulations of PerC [40], and the schematic diagram and on-site measurement device are shown in Figure 1.

The theoretical basis of this measuring device was Darcy’s law, in which the water head was determined by controlling the water level difference at the two opening positions of the square cylinder and overflow tank, as shown in Figure 1b. The measurement steps of the permeability coefficient were as follows:

1. Sample processing. Except for the permeable surface of the sample, all other surfaces were sealed with structural adhesive to prevent water from penetrating from the sides during the test. After the sealant solidified, the sample was immersed in water for 20 min and then removed for use.
2. Sample installation. The processed specimen was inserted into the square cylinder (part 3 in Figure 1) and then the base was assembled (part 10 in Figure 1) at the bottom of the square cylinder, as shown in Figure 1c. Then, the square cylinder was placed into the water tank (part 4 in Figure 1) and the upper edge of the specimen was sealed again with rubber putty.
3. Permeability test. The water supply system was opened to allow water to enter the square cylinder. When water flowed out from the overflow port of the water tank (part 5 in Figure 1), the water supply was adjusted to stabilize the water flow from the overflow port of the water tank and square cylinder. Then, a measuring cylinder was used to collect water from the overflow port of the water tank and the water flow was recorded for 5 min.
4. Calculation of permeability coefficient. The permeability coefficient was calculated according to Formula (1):

(1) $$K=\frac{QL}{AHt}$$

where K (mm/s) is the permeability coefficient of the specimen, Q (mm^3^) is the amount of water flowing from the overflow port of the water tank in t seconds, L (mm) is the thickness of the specimen, A (mm^2^) is the surface area of the specimen, H (mm) is the water level difference, and t (s) is the time.

The permeability test was repeated three times for each specimen, and the permeability coefficient was averaged from the results of the three tests.

## 3. Results and Discussion

### 3.1. Comparison of Bottom Surface Morphology of PRAC Specimens

**Figure 2:**
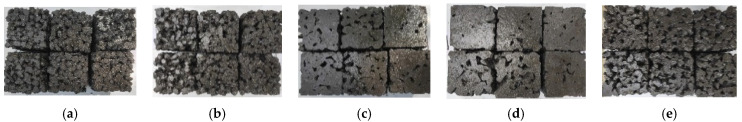
Comparison of bottom surface morphology of pervious concrete with different RCA replacement rates (vibration molding for 5 s): (**a**) group 02 (without RCA), (**b**) group A2 (RCA replacement: 30%), (**c**) group B2 (RCA replacement: 50%), (**d**) group C2 (RCA replacement: 70%), and (**e**) group 04 (RCA replacement: 100%).

The permeability of pervious concrete is primarily influenced by the settling of slurry at the bottom and the pore area on the bottom surface of specimens. A comparison of the bottom surface morphology of PerC with various RCA dosages is shown in Figure 2, where a coarse aggregate with particle sizes of 9.5–16 mm was used and subjected to vibration molding for 5 s.

It was evident that when the RCA replacement did not exceed 70%, the bottom sedimentation slurry of PRAC increased and the bottom pores decreased as the RCA replacement increased. On the one hand, this was due to the fact that the RCA was pre-wetted before casting the PRAC to solve the problem of the higher water absorption of RCA, so that the cement slurry produced by the hydration reaction increased with the increase in the RCA substitution rate and the slurry sedimentation phenomenon induced by mechanical vibration became more obvious. On the other hand, when replacing NCA with RCA of equal weight in the mix design, we observed that the higher the RCA replacement rate, the less cement content per unit volume of concrete, which could not be ignored.

**Figure 3:**
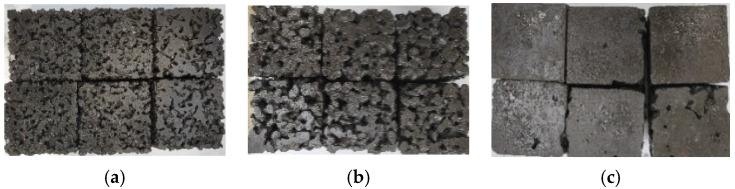
Comparison of bottom surface morphology of PRAC with different aggregate particle sizes (vibration molding for 5 s): (**a**) group 03 (aggregate particle sizes: 4.75~9.5 mm), (**b**) group 04 (aggregate particle sizes: 9.5~16 mm), and (**c**) group 05 (aggregate particle sizes: 16~19 mm).

Figure 3 illustrates a comparison of the effect of the aggregate particle size on the morphology of the bottom surface of PRAC under conditions of 100% RCA replacement and vibration molding for 5 s.

It could be seen that the pores on the bottom surface of PRAC made with aggregate particle sizes of 4.75~9.5 mm were the most numerous and homogeneous but the pores on the bottom surface of PRAC made with aggregate particle sizes of 16~9 mm were significantly reduced, which indicated that the number of pores on the bottom surface of the PRAC specimens decreased with an increase in the aggregate particle size. This phenomenon occurred because the smaller the particle size of the coarse aggregate within the same cross-section of PRAC, the greater the amount of coarse aggregate it contained, resulting in a greater number of pores between the aggregates. Additionally, there was a significant difference in the pore morphology at the bottom of the PRAC when the aggregate particle sizes were 9.5~16 mm or 16~19 mm, respectively, due to the fact that the differences in the aggregate particle sizes in both cases were 6.5 mm and 3 mm, respectively, and the larger difference in the particle sizes allowed for more smaller particles to fill the pore spaces between the larger particles.

**Figure 4:**
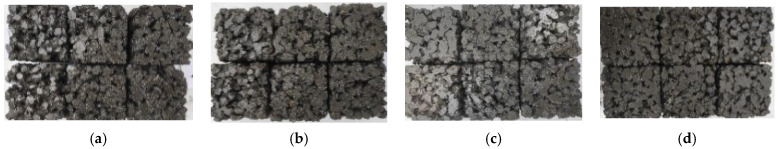
Comparison of bottom surface morphology of PRAC produced using different forming methods: (**a**) group A2 (vibration forming: 5 s), (**b**) group D1 (vibration forming: 10 s), (**c**) group D2 (vibration forming: 15 s), and (**d**) group D3 (layered tamping).

Figure 4 shows the effects of different molding methods on the bottom surface morphology of PRAC under the condition of a 30% RCA substitution rate and aggregate particle sizes of 9.5~16 mm.

As the mechanical vibration time increased, the effect of slurry settling on the bottom surface of the PRAC became more obvious, resulting in fewer pores, which indicated that vibration molding within 15 s did not excessively cause segregation between the slurry and the aggregate. In addition, there was no significant difference in the bottom surface morphology of PRAC specimens produced by vibration molding and layered insertion-tamping, respectively.

### 3.2. The Effect of RCA Replacement on the Performance of Pervious Concrete

**Figure 5:**
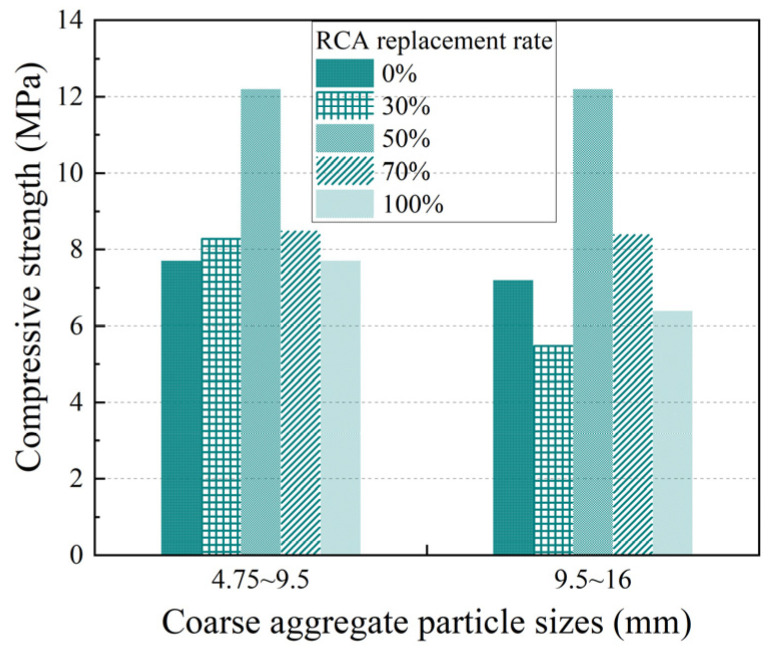
Effect of RCA replacement on compressive strength of pervious concrete.

**Figure 6:**
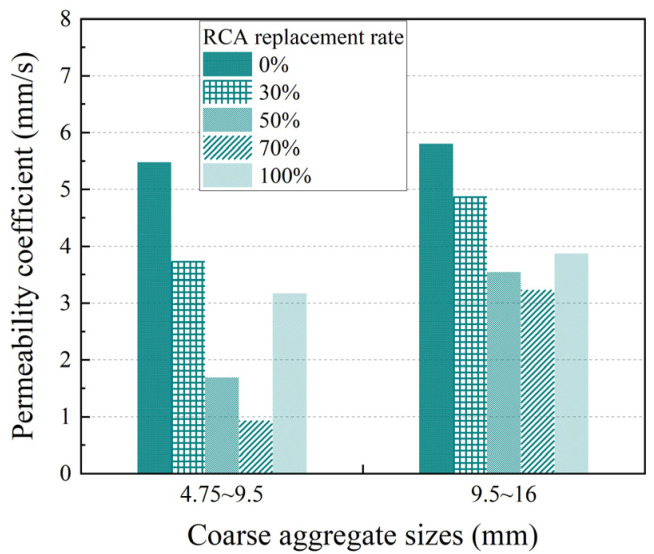
Effect of RCA replacement on permeability coefficient of pervious concrete.

Figure 5 and Figure 6 show the influence of the RCA replacement rate on the compressive strength and permeability coefficient, respectively, for the two types of PerC containing coarse aggregates with particle sizes of 4.75 mm~9.5 mm or 9.5 mm~16 mm.

With an increase in the RCA substitution rate, the compressive strength of pervious concrete showed a trend of first increasing and then decreasing, while the permeability coefficient tended to decrease and then increase, which was similar to most research conclusions [17,18]. For example, in the case of aggregate particle sizes of 4.75~9.5 mm, the compressive strength of PRAC with a 30% or 50% RCA replacement increased by 7.8% or 58.4% compared with the control group (test group 01), while the compressive strength of PRAC with a 70% or 100% RCA replacement decreased by 30.3% or 36.9% compared with that with a 50% RCA replacement, respectively. The maximum compressive strength of PRAC was achieved at a 50% RCA replacement. In the case of aggregate particle sizes of 9.5~16 mm, the permeability coefficient of PRAC with 30%, 50%, and 70% RCA substitution decreased by 15.9%, 39.0%, and 44.1%, respectively, compared with the control group (test group 02). The permeability coefficient of PRAC with 100% RCA substitution increased by 19.4% compared with that of PRAC with 70% RCA substitution.

**Figure 7:**
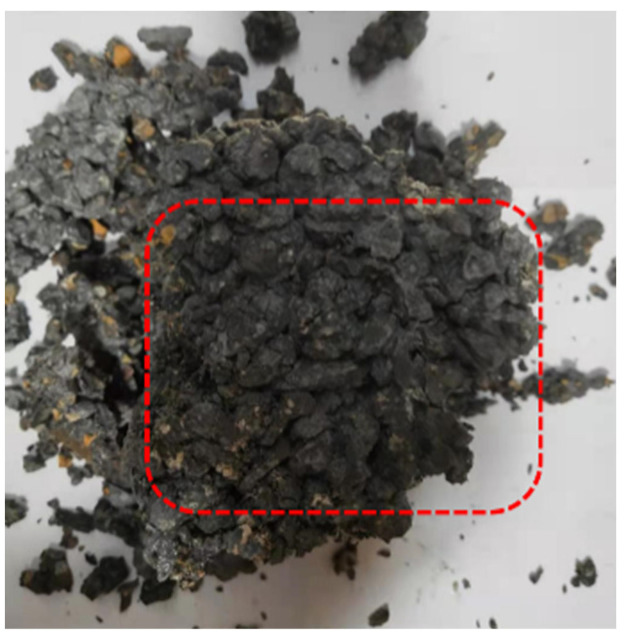
Crushed form of pervious concrete.

The compressive strength of pervious concrete, due to its higher internal porosity, mainly derives from the contact area between the coarse aggregates, and the primary mode of failure is slurry breaking between these aggregates (as shown in Figure 7).

With an increase in the RCA substitution in PRAC, the mixing water absorbed by the pre-wetted RCA decreased, leading to an increase in the cement slurry around the coarse aggregates, which resulted in an increase in the compressive strength and a decrease in the permeability coefficient. On the other hand, when replacing NCA with RCA of equal weight, as mentioned earlier, the amount of cement per unit volume decreased as the RCA replacement ratio increased. It was the result of the combined action of these two aspects. However, an excessive RCA substitution adversely affects the strength of PRAC, which is mainly due to the presence of old cement paste on the surface of RCA that is more brittle than the natural aggregate and the existence of a weak interfacial transition zone between RCA and old cement paste.

### 3.3. The Effect of Aggregate Size on the Performance of Pervious Concrete

**Figure 8:**
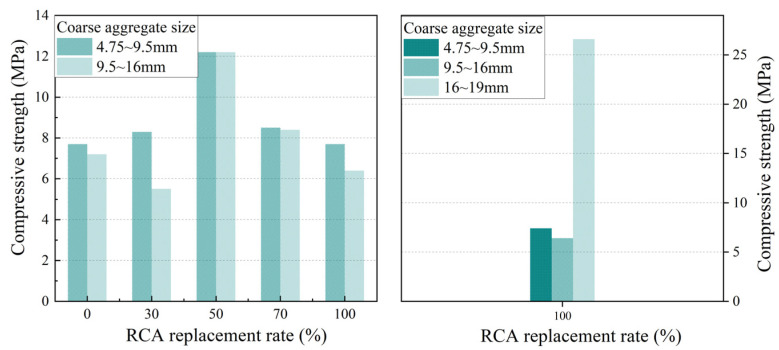
Effect of coarse aggregate size on the compressive strength of PRAC.

**Figure 9:**
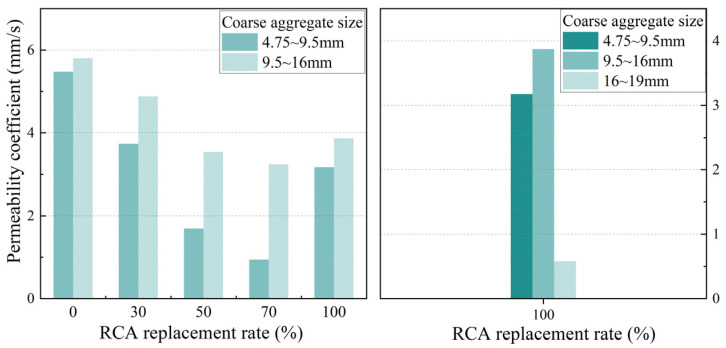
Effect of coarse aggregate size on the permeability coefficient of PRAC.

Based on data obtained from experimental measurements, the influence of the coarse aggregate particle size on the compressive strength and permeability coefficient of PRAC with various RCA substitution rates and a 5 s vibration molding process is presented in Figure 8 and Figure 9, respectively.

Pervious concrete with coarse aggregate particle sizes of 4.75~9.5 mm exhibits a higher compressive strength and lower permeability coefficient than that with coarse aggregate particle sizes of 9.5~16 mm. Furthermore, pervious concrete featuring coarse aggregate particle sizes of 16~19 mm demonstrates superior compressive strength and a reduced permeability coefficient when contrasted with the former two cases, indicating that the influence of aggregate particle size on the performance of PRAC cannot be ignored [20,21]. For example, when the RCA substitution rates were 0%, 30%, and 100%, respectively, the compressive strength of pervious concrete with particle sizes of 9.5~16 mm decreased by 6.5%, 32.5%, and 16.9% compared with those with particle sizes of 4.75~9.5 mm. Concurrently, the increases in the permeability coefficient were 5.8%, 30.5%, and 22.1%, respectively. In the case of the 100% RCA substitution rate, the compressive strength of the PRAC with coarse aggregate particle sizes of 16~19 mm increased by 245.5% and 315.6% and the permeability coefficient decreased by 81.7% and 85% compared with those with particle sizes of 4.75~9.5 mm and 9.5~16 mm, respectively.

**Figure 10:**
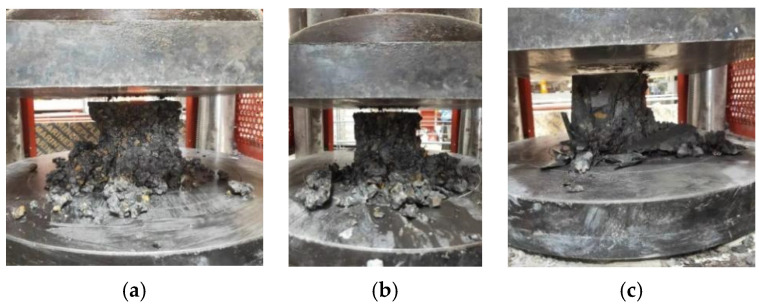
Crushing morphology of concrete with different coarse aggregate particle sizes: (**a**) 4.75~9.5 mm, (**b**) 9.5~16 mm, and (**c**) 16~19 mm.

The crushing behavior of PerC differs from that of ordinary concrete, which typically exhibits central cracking. Instead, PerC undergoes overall irregular crushing due to relative slippage between individual coarse aggregates, as depicted in Figure 10.

The compressive strength of pervious concrete primarily derives from the contact stress between the coarse aggregates and the bonding stress of the binding material. As the particle size per unit volume decreases, the number of particles increases. Consequently, the total contact area in the pervious concrete with particle sizes of 4.75~9.5 mm was greater than that in the concrete with particle sizes of 9.5~16 mm, resulting in higher compressive strength for the former but a lower permeability coefficient. In the case of pervious concrete with coarse aggregate sizes of 16~19 mm, the inter-aggregate pore area was the largest and through pores were more abundant. After the vibration molding process, the cement paste filled these pores more effectively, leading to a significant increase in strength compared with the previous two scenarios. However, this increase in strength came at the cost of significantly reduced permeability. From the crushed concrete surface depicted in Figure 10c, it was evident that the cement slurry underwent almost complete crushing, with the through pores on the crushed surface being almost entirely filled by the cement paste, while there was relatively little coarse aggregate.

### 3.4. Influence of Molding Method on the Performance of PRAC

**Figure 11:**
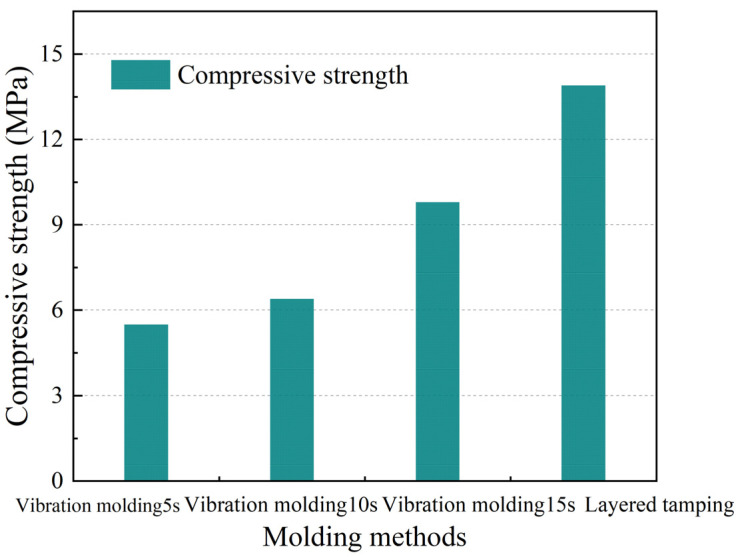
Effect of molding methods on the compressive strength of PRAC.

**Figure 12:**
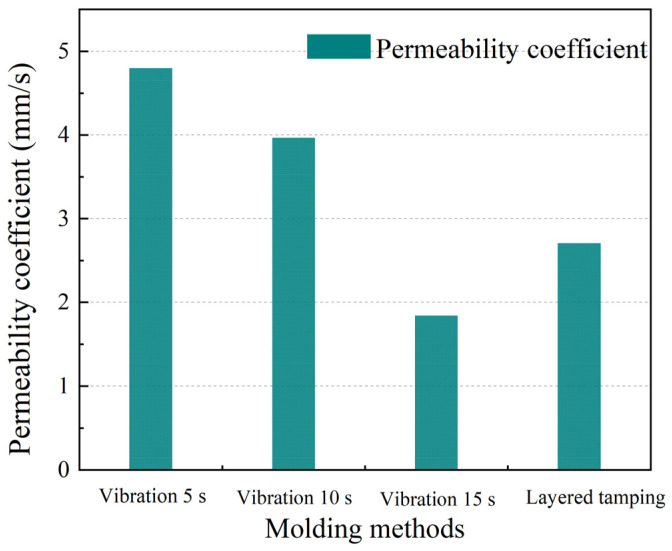
Effect of molding methods on the permeability coefficient of PRAC.

The effects of different molding methods on the compressive strength and permeability coefficient of PRAC with an RCA substitution rate of 30% and coarse aggregate particle sizes of 9.5~16 mm are shown in Figure 11 and Figure 12, respectively.

The compressive strength of the PRAC prepared using the vibration molding method gradually increased with an increase in the vibration time but the permeability coefficient gradually decreased. PerC has a much higher porosity than ordinary concrete due to a lack of fine aggregates [4,5]. The longer the vibration time during the vibration molding process, the more likely the liquid cement slurry will float up and easily block the pores (the denser the concrete becomes), resulting in an increase in compressive strength but a decrease in permeability.

In addition, the compressive strength of the PRAC prepared using the layered insertion-tamping method was higher than that of the vibration molding method. For example, the compressive strength of PRAC produced by layered insertion-tamping was 41.2% higher than that produced by vibration molding with a maximum vibration time of 15 s and the permeability coefficients were reduced by 44.5% and 31.7%, respectively, compared with PRAC produced by vibration molding for 5 s and 10 s, but increased by 7.3% compared with PRAC produced by vibration molding for 15 s. It should be noted that extending the vibration time (such as 15 s of vibration) may increase the strength of PRAC but it can also cause the permeability of PRAC to be even lower than that of PRAC prepared using the layered insertion-tamping method.

### 3.5. Mix Proportion and Molding Method of PRAC

The compressive strength and permeability coefficient measured using experiments can be used as random variables to calculate their statistical parameters such as the average value μ, standard deviation σ, and dispersion coefficient cv, as shown in Table 4. It can be seen that there was not much difference in the dispersion coefficient of compressive strength and permeability coefficient but the dispersion degree was relatively large, indicating that the substitution rate of recycled aggregates, aggregate particle size, and forming method of concrete had significant impacts on the compressive strength and permeability coefficient of PRAC.

**Figure 13:**
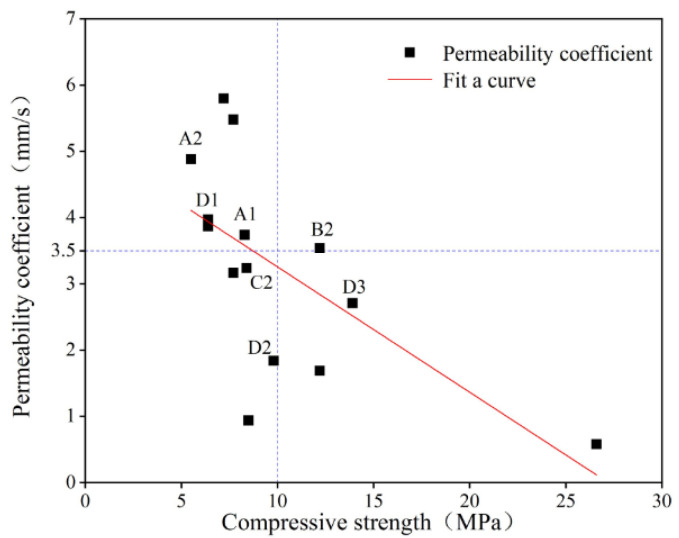
Relationship between compressive strength and permeability coefficient of PRAC.

Through the analysis of the experimental data in this study, the optimal mix proportion and molding method of PRAC under the given experimental conditions were sought. The compressive strength and permeability coefficient of PRAC are presented in Figure 13.

It was evident that the compressive strength and permeability coefficient of PRAC showed an overall inverse trend. When the internal porosity of PRAC was large, the compressive strength mainly depending on the bonding stress between aggregates was small and an increase in internal permeable pores increased the permeability coefficient (mainly determined by through pores). However, when the internal porosity of PRAC was small, the compressive strength mainly depending on the bonding stress between the aggregates and the cement slurry around the aggregates was high and a reduction in internal through pores led to a decrease in the permeability coefficient.

Pervious concrete that meets practical engineering applications needs to have both good permeability and sufficient compressive strength. Under the premise of considering both the compressive strength and permeability coefficient, it was necessary to select and optimize a set of optimal mix proportions. For the PRAC studied in this article, the permeability coefficient of the PRAC was mainly distributed between 1~6 mm/s and the compressive strength was mainly distributed between 5~15 MPa. The two dashed lines in Figure 13 represent the median values of the distribution intervals of the permeability coefficient and compressive strength (3.5 mm/s and 10 MPa, respectively). Here, if the evaluation was simply that the compressive strength and permeability coefficient of PRAC were not lower than the median values of the experimental data, it was obvious that only test group B2 in Table 3 met the requirements. Considering the compressive strength and permeability of PRAC comprehensively, the PRAC with the RCA substitution rate of 50% and coarse aggregate sizes of 9.5~16 mm prepared by vibration molding for 5 s had relatively good permeability and compressive strength.

## 4. Conclusions

This study primarily investigated the impacts of the RCA substitution rate, particle size of coarse aggregates, and molding methods on the strength and permeability of PRAC, with the aim of determining the optimal mix ratio and molding method for PRAC to have sufficient compressive strength and good permeability. The main conclusions were as follows:

1. With an increase in the RCA substitution rate, the compressive strength of PRAC showed a trend of first increasing and then decreasing, and reached its maximum at a 50% RCA substitution rate. Adding RCA decreased the permeability of PerC.
2. The influence of coarse aggregate size on PRAC cannot be ignored. Although larger coarse aggregate sizes (such as 16~19 mm) can significantly increase the compressive strength of PRAC, the corresponding permeability coefficient will significantly decrease.
3. For the vibration molding method of preparing PRAC, prolonging the vibration time increases the strength of PRAC and reduces its permeability. In addition, the layered insertion-tamping method enables PRAC to achieve a higher strength than the vibration molding method.
4. Given the overall inverse trend of the compressive strength and permeability coefficient of PRAC under the experimental conditions presented in this article, an RCA substitution rate of 50%, coarse aggregate sizes of 9.5~16 mm, and vibration molding for 5 s can enable PRAC to have both relatively good permeability and strength.

## Figures and Tables

**Table 1:** Chemical composition and physical properties of Portland cement.

Chemical Composition		Physical Properties	
Composition	Content/%	Category	Measured Value
CaO	55.49	Specific surface area/m^2^/kg	362
SiO_2_	22.94	Standard consistence/%	28
Al_2_O_3_	8.5	Loss on ignition/%	3.06
Fe_2_O_3_	3.52	Initial setting time/min	155
MnO	1.75	Final setting time/min	239
MgO	1.75	3-day flexural strength/MPa	5.4
SO_3_	1.62	3-day compressive strength/MPa	25.3
K_2_O	0.62	28-day flexural strength/MPa	8.6
TiO_2_	0.35	28-day compressive strength/MPa	49.8
Na_2_O	0.18		

**Table 2:** Physical properties of recycled aggregate and natural aggregate.

Aggregate Type	Performance Index			
	Apparent Density (kg/m^3^)	Crush Index (%)	Bulk Density (g/cm^3^)	Water Absorption (%)
RCA	2670	18.4	1.305	3.93
NCA	2731	11.5	1.632	1.78

**Table 3:** Mix proportions of PRAC per unit volume.

Test Group Number	Molding Method	RCA Replacement/%	Aggregate Particle Size/mm	Cement /kg	NCA /kg	RCA /kg	Water /kg	Water Reducer/g
01	Vibration molding, 5 s	0	4.75~9.5	334.7	1599.4	0	100.4	1673.5
02			9.5~16	334.7	1599.4	0	100.4	1673.5
A1		30	4.75~9.5	334.7	1119.6	479.8	100.4	1673.5
A2			9.5~16	334.7	1119.6	479.8	100.4	1673.5
B1		50	4.75~9.5	334.7	799.7	799.7	100.4	1673.5
B2			9.5~16	334.7	799.7	799.7	100.4	1673.5
C1		70	4.75~9.5	334.7	479.8	1119.6	100.4	1673.5
C2			9.5~16	334.7	479.8	1119.6	100.4	1673.5
03		100	4.75~9.5	334.7	0	1599.4	100.4	1673.5
04			9.5~16	334.7	0	1599.4	100.4	1673.5
05			16~19	334.7	0	1599.4	100.4	1673.5
D1	Vibration molding,10 s	30	9.5~16	334.7	1119.6	479.8	100.4	1673.5
D2	Vibration molding, 15 s			334.7	1119.6	479.8	100.4	1673.5
D3	Layered insertion-tamping			334.7	1119.6	479.8	100.4	1673.5

**Table 4:** Statistical parameters of compressive strength and permeability coefficient.

Random Variables	Average Value *μ*	Standard Deviation *σ*	$\mathrm{Dispersion}\;\mathrm{Coefficient}\;c_{v}=\sigma /\mu$
Compressive strength (MPa)	10.06	5.35	0.53
Permeability coefficient (mm/s)	3.25	1.58	0.49

## Data Availability

The original contributions presented in the study are included in the article, further inquiries can be directed to the corresponding authors.

## References

[1] Debnath B., Sarkar P.P. (2019). Permeability prediction and pore structure feature of pervious concrete using brick as aggregate. Constr. Build. Mater..

[2] Alshareedah O., Nassiri S. (2021). Pervious concrete mixture optimization, physical, and mechanical properties and pavement design: A review. J. Clean. Prod..

[3] Yang Y.F. (2023). Research on the Influence of Pore Characteristics on the Performance of Permeable Concrete. Master’s Thesis.

[4] Elango K.S., Gopi R., Saravanakumar R., Rajeshkumar V., Vivek D., Raman S.V. (2021). Proceedings Properties of pervious concrete– A state of the art review. Mater. Today Proc..

[5] Lima G.T.D.S., Rocha J.C., Cheriaf M. (2022). Investigation of the properties of pervious concrete with a recycled aggregate designed with a new combination of admixture. Constr. Build. Mater..

[6] Debnath B., Sarkar P.P. (2020). Pervious concretes an alternative pavement strategy: A state-of-the-art review. Int. J. Pavement Eng..

[7] Liang X., Cui S., Li H., Abdelhady A., Wang H., Zhou H. (2019). Removal effect on stormwater runoff pollution of porous concrete treated with nanometer titanium dioxide. Transp. Res. Part D Transp. Environ..

[8] Zhang X.H., Meng Y.F., Ren J. (2013). Preliminary study of the present situation and development for the recycled aggregate concrete in domestic and foreign. Concrete.

[9] Yang H., Xia J., Thompson J.R., Flower R.J. (2017). Urban construction and demolition waste and landfill failure in Shenzhen, China. Waste Manag..

[10] Tam V.W.Y., Soomro M., Evangelista A.C.J. (2018). A review of recycled aggregate in concrete applications (2000–2017). Constr. Build. Mater..

[11] Thomas J., Thaickavil N.N., Wilson P.M. (2018). Strength and durability of concrete containing recycled concrete aggregates. J. Build. Eng..

[12] Cakir O. (2014). Experimental analysis of properties of recycled coarse aggregate (RCA) concrete with mineral additives. Constr. Build. Mater..

[13] Xiao J., Li W., Sun Z., Lange D.A., Shah S.P. (2013). Properties of interfacial transition zones in recycled aggregate concrete tested by nanoindentation. Cem. Concr. Compos..

[14] Guneyisi E., Gesoglu M., Kareem Q., İpek S. (2016). Effect of different substitution of natural aggregate by recycled aggregate on performance characteristics of pervious concrete. Mater. Struct..

[15] Sata V., Wongsa A., Chindaprasirt P. (2013). Properties of pervious geopolymer concrete using recycled aggregates. Constr. Build. Mater..

[16] Barnhouse P.W., Srubar W.V. (2016). Material characterization and hydraulic conductivity modeling of macroporous recycled-aggregate pervious concrete. Constr. Build. Mater..

[17] Zaetang Y., Sata V., Wongsa A., Chindaprasirt P. (2016). Properties of pervious concrete containing recycled concrete block aggregate and recycled concrete aggregate. Constr. Build. Mater..

[18] Chen S., Chang C., Guo L., Xue Z. (2018). Influence of recycled aggregates content on the performance of pervious concrete. J. Basic Sci. Eng..

[19] Park S.B., Lee B.J., Lee J., Jang Y.I. (2010). A study on the seawater purification characteristics of water-pervious concrete using recycled aggregate. Resour. Conserv. Recycl..

[20] Yuan H.Q., Jiang Y.B., Cui Y.L., Zhou H. (2018). Permeability and Compressive Strength of Recycled Aggregate Permeable Concrete. Mater. Rep..

[21] Gao M.Y. (2014). The Experimental Study of Recycled Aggregate Pervious Concrete. Master’s Thesis.

[22] Yan H.D., Huang G.H. (2006). Study on Pervious Road Brick Prepared by Recycled Aggregate Concrete. Key Eng. Mater..

[23] Xue J., Liu J., Ji M.X., Zou Z.P., Shi H.B., Cao H. (2012). Preparation of permeable bricks using recycled architectural concrete as aggregate. J. Wuhan Inst. Technol..

[24] Li J., Liu Z. (2011). Microanalysis of Recycled Coarse Aggregate and Properties of No-Fines Pervious Recycled Concrete. J. Test. Eval..

[25] Liu F.Y. (2012). Studies on Ecological Pervious Brick by making use of Construction Waste. Master’s Thesis.

[26] Gaedicke C., Marines A., Miankodila F. (2014). Assessing the abrasion resistance of cores in virgin and recycled aggregate pervious concrete. Constr. Build. Mater..

[27] Hesami S., Ahmadi S., Nematzadeh M. (2014). Effects of rice husk ash and fiber on mechanical properties of pervious concrete pavement. Constr. Build. Mater..

[28] Zhang H.B., Du X.Q., Kou J.L., Yu D.H. (2017). Experimental Study on Compressive and Permeable Performance of Pervious Concrete with Recycled Aggregates. J. Exp. Mech..

[29] Guo L., Liu S.Y., Chen S.K., Wang L.Y., Xue Z.L. (2019). Research on mechanical properties, permeability and abrasion resistance of fibers modified recycled aggregate pervious concrete. Trans. Chin. Soc. Agric. Eng..

[30] Xue D.J., Liu R.G., Xu R.J., He Z.Q. (2013). Preparation and basic charact eristics for pervious concrete made from recycled aggregate. Concrete.

[31] (2016). Technical Specification for Application of Pervious Recycled Aggregate Concrete.

[32] (2007). Common Portland Cement.

[33] (2006). Standard for Technical Requirements and Test Method of Sand and Crushed Stone (or Gravel) for Ordinary Concrete.

[34] (2011). Technical Specification for Application of Recycled Aggregate.

[35] (2010). Recycled Coarse Aggregate for Concrete.

[36] (2017). Polycarboxylates High Performance Water-Reducing Admixture.

[37] (2006). Standard of Water for Concrete.

[38] (2011). Code for Design of Recycled Concrete Structures.

[39] Xiao J., Li J., Sun Z., Hao X. (2004). Study on compressive strength of recycled aggregate concrete. J. Tongji Univ. Nat. Sci..

[40] (2009). Technical Specification for Pervious Cement Concrete Pavement.

[41] (2009). Standard for Test Methods of Concrete Physical and Mechanical Properties.

